# Facilitating person‐centred care: integrating an electronic client feedback tool into continuous quality improvement processes to deliver client‐responsive HIV services in the Democratic Republic of Congo

**DOI:** 10.1002/jia2.26112

**Published:** 2023-07-06

**Authors:** Cyprien Tendo‐Bugondo, Oséé Lieke, Pierre Kasongo, Baudouin Diur, Davina S. Canagasabey, Ibou Thior, Pascal K. Milenge, Jean‐Claude Kiluba

**Affiliations:** ^1^ PATH Lubumbashi Democratic Republic of the Congo; ^2^ Independent Consultant Lubumbashi Democratic Republic of the Congo; ^3^ Macro‐Eyes Kinshasa Democratic Republic of the Congo; ^4^ Union Congolaise des Organisations des Vivant avec VIH Lubumbashi Democratic Republic of the Congo; ^5^ PATH Washington DC USA

**Keywords:** person‐centred care, quality improvement, client feedback, digital tools, peer‐led, HIV

## Abstract

**Introduction:**

Engaging communities in the design, implementation and monitoring of health services is critical for delivering high‐quality, person‐centred services that keep people living with HIV engaged in care. The USAID‐funded Integrated HIV/AIDS Project in Haut‐Katanga (IHAP‐HK) integrated an electronic client feedback tool into continuous quality improvement (CQI) processes. We aimed to demonstrate this system's impact on identifying and improving critical quality‐of‐care gaps.

**Methods:**

Through stakeholder and empathy mapping, IHAP‐HK co‐designed a service quality monitoring system—comprising anonymous exit interviews and ongoing monitoring through CQI cycles—with people living with HIV, facility‐based providers and other community stakeholders. IHAP‐HK trained 30 peer educators to administer oral, 10‐ to 15‐minute exit interviews with people living with HIV following clinic appointments, and record responses via the KoboToolbox application. IHAP‐HK shared client feedback with facility CQI teams and peer educators; identified quality‐of‐care gaps; discussed remediation steps for inclusion in facility‐level improvement plans; and monitored implementation of identified actions. IHAP‐HK tested this system at eight high‐volume facilities in Haut‐Katanga province from May 2021 through September 2022.

**Results:**

Findings from 4917 interviews highlighted wait time, stigma, service confidentiality and viral load (VL) turnaround time as key issues. Solutions implemented included: (1) using peer educators to conduct preparatory tasks (pre‐packaging and distributing refills; pulling client files) or escort clients to consultation rooms; (2) limiting personnel in consultation rooms during client appointments; (3) improving facility access cards; and (4) informing clients of VL results via telephone or home visits. Due to these actions, between initial (May 2021) and final interviews (September 2022), client satisfaction with wait times improved (76% to 100% reporting excellent or acceptable wait times); reported cases of stigma decreased (5% to 0%); service confidentiality improved (71% to 99%); and VL turnaround time decreased (45% to 2% informed of VL results 3 months after sample collection).

**Conclusions:**

Our results showed the feasibility and effectiveness of using an electronic client feedback tool embedded in CQI processes to collect client perspectives to improve service quality and advance client‐responsive care in the Democratic Republic of Congo. IHAP‐HK recommends further testing and expansion of this system to advance person‐centred health services.

## 1 INTRODUCTION

Comprehensive community engagement is critical for delivering high‐quality, person‐centred care (PCC) that supports the continued engagement of people living with HIV in care and facilitates better health outcomes. PCC is critical to achieving high quality of care, improving clinical outcomes and promoting quality of life [1], with Berwick noting that “the experience of patients” should serve as “the fundamental source of the definition of quality [2].” Understanding patient satisfaction is increasingly recognized as critical for improving continuity in HIV care and enhanced outcomes [3, 4], especially in the context of differentiated HIV service models [5]. Client feedback mechanisms are included in HIV‐PCC frameworks, with the literature noting “client feedback mechanisms [to be] integral components…and hold potential for people to shape services based upon their own needs [6, 7].” Numerous studies have also highlighted the efficacy of using continuous quality improvement (CQI) to improve healthcare quality [8], including to promote person‐centred HIV services [9, 10, 11].

We consider person‐centred HIV care to be HIV services that are informed by and respect the expressed preferences of people living with HIV. Through the US Agency for International Development‐funded Integrated HIV/AIDS Project in Haut‐Katanga (IHAP‐HK) in the Democratic Republic of Congo (DRC), we aimed to co‐design a client feedback tool with people living with HIV and healthcare providers and deploy it as part of a service quality monitoring system to facilitate person‐centred HIV services at project‐supported facilities.

This paper describes the electronic client feedback tool and its integration into CQI processes to ensure facility‐level service delivery and quality improvement (QI) initiatives reflect client perspectives. It aims to demonstrate the feasibility and effectiveness of this service quality monitoring system in identifying and addressing quality‐of‐care gaps not aligned with client needs and preferences.

## 2 METHODS

### 2.1 Application of human‐centred design to create feedback tool

IHAP‐HK applied human‐centred design, using PATH's Living Labs approach [12], to co‐create a service quality monitoring system to ensure that community priorities drove intervention design. Using stakeholder mapping to ensure a representative sampling of perspectives was included during design, IHAP‐HK convened 20 individuals (five community‐based organization representatives; four facility‐based providers; eight people living with HIV; two DRC Ministry of Health representatives; and one religious leader) to co‐design a client feedback tool.

The group opted to use an anonymous, electronic exit interview to gather client feedback. IHAP‐HK used empathy mapping [13]—a process for gathering user insight on their experiences with services or products—with stakeholders to identify frequent pain points encountered by clients and prioritize service delivery aspects the exit interview should focus on. Empathy maps are created in early design stages, following initial research and before ideation, to help design teams better understand clients being reached [14].

Stakeholders drafted interview questions in small groups, with draft questions finalized together in a large group and validated by the MOH. The final questionnaire comprised the following questions:

- **Wait time**: How long did you wait before being received by a healthcare provider?
- **Medication dispensing**: Did you receive all prescribed medications?
- **Provider attitude**: What was the provider's attitude towards you? Did you feel stigmatized by facility staff? Were services offered to you in complete confidentiality?
- **Viral load (VL) services**: Have you had a VL sample taken in the past 6 months? Did you receive your results, and if so, how long did it take to receive your results?
- **Recommendations**: Do you have suggestions to improve the quality of services received?

The interview questionnaire was programmed in French and Swahili into the KoBoToolbox digital application (an open‐source data collection tool used in low‐resource environments). Thirty people living with HIV serving as peer educators (70% female) were trained to conduct exit interviews (ranging 10–15 minutes) and record responses into the application using project‐supplied tablets or phones.

### 2.2 Incorporation into CQI processes

**Figure 1:**
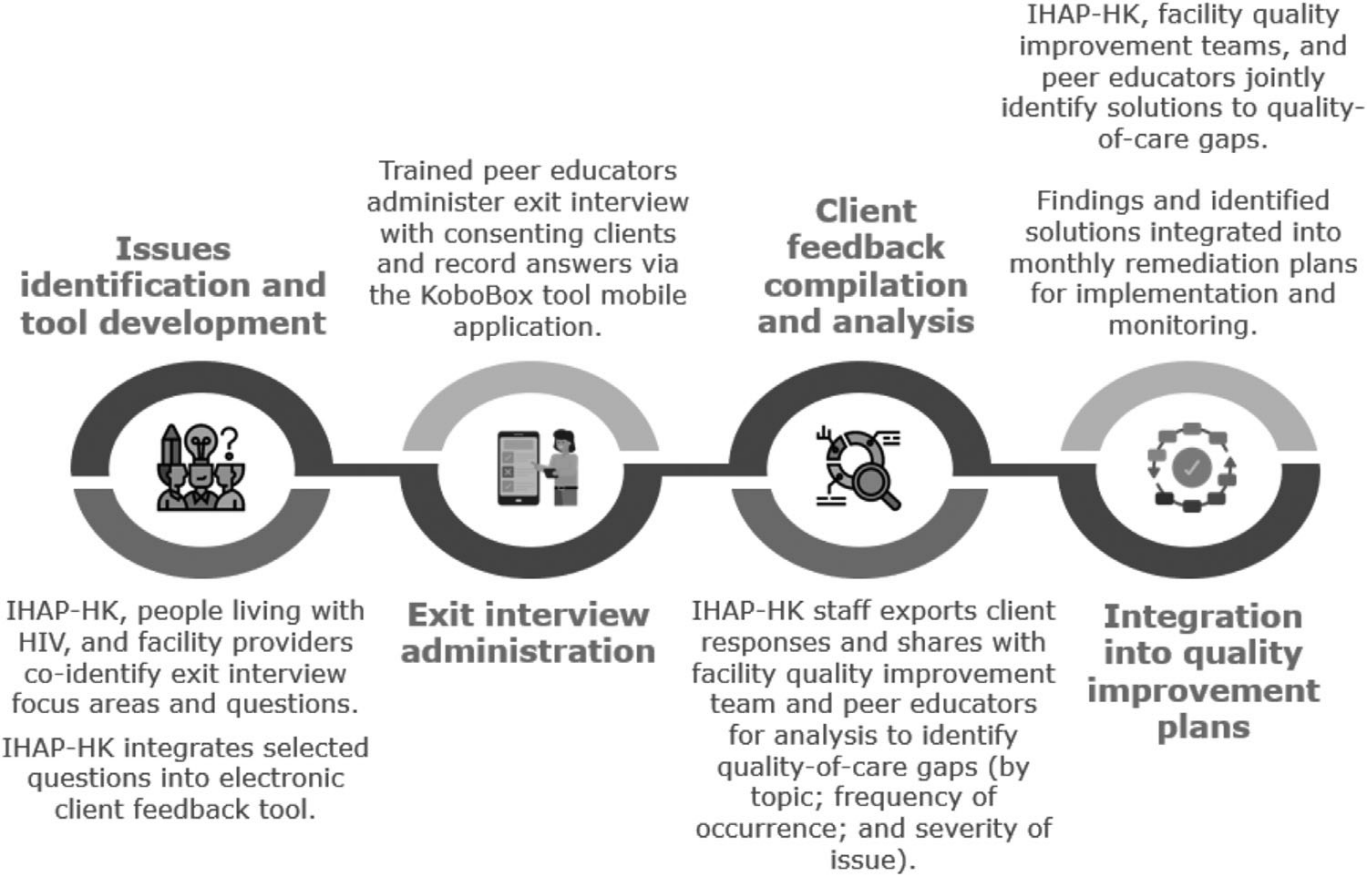
Service quality monitoring feedback loop. Abbreviation: IHAP‐HK, International Development‐funded Integrated HIV/AIDS Project in Haut‐Katanga.

To ensure that issues raised by clients were systematically addressed, we embedded the electronic client feedback tool into IHAP‐HK's facility‐level CQI system. Figure 1 highlights how the tool was integrated into project CQI processes to create a continuous service monitoring feedback loop. IHAP‐HK staff shared client feedback with facility QI teams and peer educators monthly to identify key quality‐of‐care challenges and client recommendations. IHAP‐HK staff, facility QI teams and peer educators then brainstormed approaches to address challenges, implemented these strategies and monitored the impact on identified challenges, following a Plan‐Do‐Study‐Act methodology [15] to iteratively assess impact through data gathered via the electronic client feedback tool and adjust solutions over time. Successful strategies were also shared among IHAP‐HK facility QI teams during quarterly learning sessions for adoption at other facilities with similar care challenges.

### 2.3 Data capture and analysis

IHAP‐HK tested this service quality monitoring system at eight project‐supported, high‐volume healthcare facilities across six health zones of Haut‐Katanga. To mitigate selection bias, all clients were offered the opportunity to provide feedback following clinic appointments. We analysed anonymous interview data collected between May 2021 and September 2022, using descriptive statistics and Stata 13.1 to compare results between initial interviews (May 2021) and final interviews (September 2022) to understand changes in perceived client satisfaction against prioritized service delivery components/questionnaire variables during system implementation (outcome measure). Our analysis included testing for equality of median waiting time distribution across exit interviews using the Kruskal‐Wallis test and recoding clients’ suggestions to identify additional service quality challenges and recommended solutions.

Client participation was voluntary, with clients invited to participate for QI purposes; individual verbal consent was obtained before interviews were administered. Ethical approval was not required as interviews were used to yield knowledge used for immediate local action through project CQI activities.

## 3 RESULTS

Four thousand nine hundred and seventeen exit interviews were conducted with clients receiving antiretroviral treatment (ART) at pilot facilities. Most clients providing feedback were female (65%) and the median age was 38 (interquartile range [29–47]), both reflective of the general composition of IHAP‐HK's ART cohort.

### 3.1 Quality gaps and QI solutions

Clients identified appointment wait times, stigma, confidentiality and VL services (specifically long turnaround times [TAT]) as the highest priority issues to be addressed at these facilities, which align with service delivery aspects that tend to be predictors of client satisfaction [16, 17, 18]. Additional recommendations included more flexible physical access to facility compounds and more welcoming waiting rooms.

IHAP‐HK, people living with HIV and facility QI teams identified and integrated the following solutions to address identified quality gaps:

- **Long wait time**: (1) leveraging peer educators to conduct appointment preparatory tasks (e.g. pulling medical records; pre‐packaging medications); (2) coaching providers to use appointment agendas to better triage needed services for clients for; (3) escorting clients to consultation rooms, pharmacies and/or laboratories; (4) improving waiting room conditions (e.g. providing chairs).
- **Reported stigma**: (1) removing HIV status from access cards used by clients to enter facilities; (2) in‐service coaching for providers on delivering services in a non‐stigmatizing manner.
- **Confidentiality**: limiting individuals permitted in consultation rooms during client appointments (e.g. essential providers; caregivers).
- **Long TAT for VL results**: (1) conducting daily follow‐up with the two referral laboratories to ensure timely analysis of IHAP‐HK client samples; (2) providing results to clients on 3‐ to 6‐month ART dispensing by short message service, telephone call or home visits, based on client preference.

### 3.2 Outcomes

Implementing QI solutions led to observed improvements to quality‐of‐care gaps between initial and final interviews.

#### 3.2.1 Wait time

Median wait time during initial interviews ranged from 5 to 9 minutes, with significant variability; the longest reported wait time was 3 hours. The mean and median wait times reported at final interviews was 7 minutes; the longest wait time was 17 minutes.

Reported client satisfaction with wait times improved, with 100% reporting wait times to be excellent or acceptable (final) compared to 76% (initial).

#### 3.2.2 Reported stigma

The percentage of clients reporting that providers’ attitudes were not good decreased between initial and final interviews, from 9% to 1%. Similarly, the percentage of clients reporting cases of stigma decreased from 5% to 0%.

#### 3.2.3 Perceptions of confidentiality

Clients reported improved confidentiality during appointments, from 71% (initial) to 99% (final).

#### 3.2.4 VL services

In May 2021, only 11 clients reported receiving their VL results following sample collection, with 45% reporting a more‐than‐3‐month TAT. In September 2022, 125 clients received their VL results, with only 2% after 3 months and 79% within 1 month, indicating decreased TAT.

## 4 DISCUSSION

The use of this client‐driven service quality monitoring system enabled IHAP‐HK to pinpoint key HIV service and quality‐of‐care gaps and rapidly deploy solutions. After applying QI solutions to identified quality‐of‐care challenges, we observed decreases in client wait time, reported stigma and VL result TAT, and increases in client satisfaction with wait time and service confidentiality. This highlights our system's effectiveness in improving HIV service quality at targeted facilities, echoing similar findings from other studies on the use of client‐driven CQI models to address service quality gaps, such as the use of Community Score Cards to improve services to prevent perinatally acquired HIV in Malawi [19], use of electronic self‐interviews integrated into rapid QI process to improve client−provider relationships in Eswatini [20] and use of CQI cycles to minimize service delays at HIV clinics in Kenya [9].

This service quality monitoring system also enabled IHAP‐HK to more rapidly flag and deploy corrective measures to address service delivery and quality issues. For example, exit interviews in early‐mid May 2021 revealed that clients in one health zone were not receiving expected ART/cotrimoxazole refills. Within 1 week, IHAP‐HK confirmed this was due to stock‐outs, requested urgent re‐supply and redistributed available stocks from nearby facilities to immediately provide clients with their medicines (compared to 1 month through normal commodity supply monitoring processes).

The use of peer educators to conduct preparatory tasks and escort clients was critical to improving the overall care experience while reducing provider administrative burden, enabling physicians and laboratory staff to provide timelier services. Our experience reinforces similar findings [21, 22] on the increased role that peer educators can play in healthcare delivery—both in service monitoring to gather client feedback and service provision by helping to refine delivery models and provide punctual, higher‐quality services.

### 4.1 Limitations

Our study had several limitations. First, the integration of this feedback tool as part of the iterative CQI methodology precluded us from isolating which aspects of our QI solutions drove our results.

Second, the use of anonymous feedback that cannot be tied back to individual clients limited exploration of patient‐level factors that may have affected service perception, such as enrolment in differentiated care and/or duration on ART. Anonymous feedback also impeded assessing the number of unique clients participating in exit interviews, meaning the same clients could be providing feedback in successive periods. As this system was devised as part of programmatic CQI efforts, we did not capture metrics to understand those who declined participation and rationale, although some clients informally noted declining due to lack of time. These aspects prevented us from gauging the true acceptability of this tool for gathering client feedback and adequately identifying potential biases.

Finally, our pilot did not assess cost‐effectiveness although measures were taken to minimize associated costs (e.g. leveraging existing peer educators; electronic [vs. paper‐based] data capture via a free app that enabled real‐time data availability and analysis through embedded visualization capabilities, which saved costs associated with manual data compilation and analysis).

With additional time, further analysis could assess the impact of implemented QI solutions on broader programmatic indicators, such as service continuity and VL coverage, to better assess the link between person‐centred approaches and HIV service delivery outcomes.

## 5 CONCLUSIONS

Our use of an electronic client feedback tool embedded in iterative CQI approaches as a client‐driven service quality monitoring system proved to be feasible and effective at rapidly identifying and deploying solutions that led to improved perception of service quality by clients receiving HIV care at eight pilot facilities in DRC. These findings indicate our system's success in highlighting HIV service delivery aspects not aligned with client needs, enabling IHAP‐HK to promote delivery models tailored to the preferences of people living with HIV. While this system holds promise to support the advancement of person‐centred HIV services to meet HIV epidemic control goals in DRC, further testing is required to inform scalability. Critical next steps will be to expand testing of this system in other provinces and compare its effectiveness (including cost‐effectiveness) with other client feedback systems used in DRC.

## COMPETING INTERESTS

The authors declare that they have no competing interests or conflicts of interest to declare.

## AUTHORS’ CONTRIBUTIONS

CT‐B, PK and OL led the creation, deployment and implementation of the electronic client feedback monitoring system, and PKM and J‐CK provided technical input and leadership throughout the process on behalf of IHAP‐HK. BD was heavily involved in co‐designing the system as a representative from an association of people living with HIV and conducted interviews as a peer educator at the Sendwe Center of Excellence. CT‐B, PK and OL oversaw data compilation, and CT‐B, OL and IT led data analysis. DSC, IT and CT‐B conceived the paper, and DSC drafted the manuscript with contributions from IT and CT‐B.

## FUNDING

Financing for the work described in this article and the IHAP‐HK project were made possible by the American people through the United States Agency for International Development (USAID CoAg. # AID‐660‐A‐17‐00001) and the US President's Emergency Plan for AIDS Relief.

## DISCLAIMER

The views expressed herein and the contents of this manuscript do not necessarily reflect the views of the United States Agency for International Development or the United States government.

## Data Availability

The data that support the findings of this study are available from the corresponding author upon reasonable request.
